# The cardiomyocyte “redox rheostat”: Redox signalling via the AMPK-mTOR axis and regulation of gene and protein expression balancing survival and death

**DOI:** 10.1016/j.yjmcc.2019.02.006

**Published:** 2019-04

**Authors:** Daniel N. Meijles, Georgia Zoumpoulidou, Thomais Markou, Kerry A. Rostron, Rishi Patel, Kenneth Lay, Balvinder S. Handa, Bethany Wong, Peter H. Sugden, Angela Clerk

**Affiliations:** a Molecular and Clinical Sciences Research Institute, St George's University of London, London SW17 0RE, UK; b School of Biological Sciences, University of Reading, Whiteknights, Reading RG6 6AS, UK; c National Heart and Lung Institute (Cardiovascular Sciences), Faculty of Medicine, Flowers Building, Imperial College, SW7 2AZ, UK and Dovehouse Street, London SW3 6LY, UK

**Keywords:** Oxidative stress, Protein synthesis, Immediate early genes, p21^Cip1/WAF1^, Cytoprotection, Raptor, mTOR, AMPK, AMP-activated protein kinase, AS, antisense, FDR, false discovery rate, IEG, immediate early gene, mTOR, mammalian target of rapamycin, mTORC, mTOR complex, ODN, oligodeoxynucleotide, ROS, reactive oxygen species.

## Abstract

Reactive oxygen species (ROS) play a key role in development of heart failure but, at a cellular level, their effects range from cytoprotection to induction of cell death. Understanding how this is regulated is crucial to develop novel strategies to ameliorate only the detrimental effects. Here, we revisited the fundamental hypothesis that the level of ROS per se is a key factor in the cellular response by applying different concentrations of H_2_O_2_ to cardiomyocytes. High concentrations rapidly reduced intracellular ATP and inhibited protein synthesis. This was associated with activation of AMPK which phosphorylated and inhibited Raptor, a crucial component of mTOR complex-1 that regulates protein synthesis. Inhibition of protein synthesis by high concentrations of H_2_O_2_ prevents synthesis of immediate early gene products required for downstream gene expression, and such mRNAs (many encoding proteins required to deal with oxidant stress) were only induced by lower concentrations. Lower concentrations of H_2_O_2_ promoted mTOR phosphorylation, associated with differential recruitment of some mRNAs to the polysomes for translation. Some of the upregulated genes induced by low H_2_O_2_ levels are cytoprotective. We identified p21^Cip1/WAF1^ as one such protein, and preventing its upregulation enhanced the rate of cardiomyocyte apoptosis. The data support the concept of a “redox rheostat” in which different degrees of ROS influence cell energetics and intracellular signalling pathways to regulate mRNA and protein expression. This sliding scale determines cell fate, modulating survival vs death.

## Highlights

• A cellular “redox rheostat” elicits different responses according to degree of redox stress.
• Low ROS levels activate mTOR, inducing protective proteins (e.g. p21^Cip1/WAF1^).
• Increasing ROS levels activate AMPK, suppressing protein synthesis via Raptor.
• High redox stress reduces ATP, inhibits protein synthesis and causes cell death.

## Graphical abstract

**Figure:**
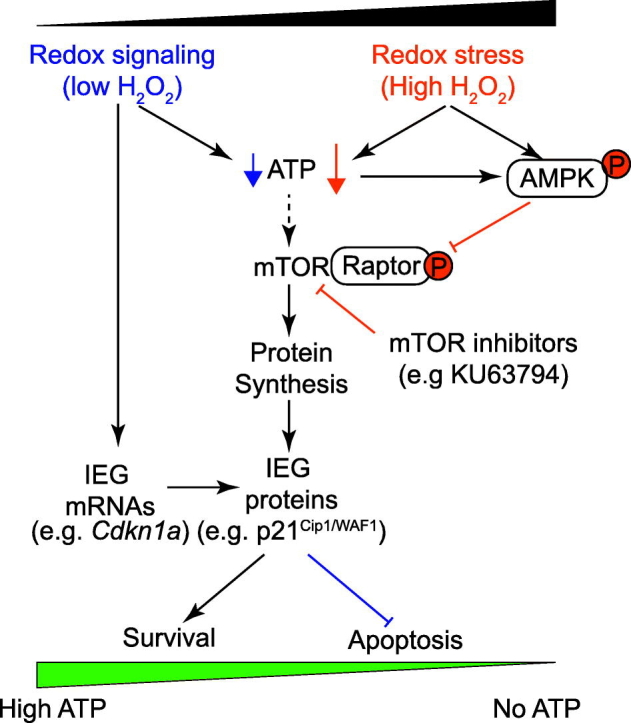

## 1 Introduction

Heart failure, the end result of various progressive heart diseases, is the leading cause of cardiovascular mortality in the developing world [1]. The consequent inability of the heart to pump blood to the body is associated with loss of terminally-differentiated contractile cardiomyocytes, whether by necrosis (as occurs following myocardial infarction) or, if energy levels suffice, one of several forms of programmed cell death including apoptosis [[2], [3], [4]]. Enhancing cardiomyocyte survival and reducing rates of cardiomyocyte death would be therapeutically advantageous, but there are no clinical strategies yet available to achieve these aims.

Oxidative stress plays a pivotal role in modulating cardiomyocyte survival, growth and death, but the effects depend on the degree of stress [5]. High levels of reactive oxygen species (ROS) are clearly damaging and result in unregulated cell death. More moderate levels induce apoptosis through the mitochondrial death pathway with cleavage and activation of caspases 9 and 3, and regulated dismantling of cellular contents. In contrast, low ROS levels are associated with cardiomyocyte survival and may even promote hypertrophy. These are examples of cellular responses by redox stress induced signalling, in which ROS activate or inhibit intracellular redox-sensitive signal transduction pathways [[6], [7], [8]]. ROS are produced either as a by-product of normal cellular metabolism (e.g. mitochondrial leakage [9]) or by dedicated ROS-producing enzymes (e.g. NADPH oxidases [10,11]). In either case, H_2_O_2_, as a stable ROS moiety, is the predominant redox effector [7], and low-level H_2_O_2_ may be vital for upregulation of pro-survival and cytoprotective genes [12].

Failing cardiomyocytes are energetically challenged and generate less ATP [13,14]. This may be due in part to increased redox stress signals. The mammalian target of rapamycin (mTOR) and AMP-activated protein kinase (AMPK) signalling pathways play a central role in responding to ATP levels to modulate cardiomyocyte function [15,16]. mTOR, an evolutionarily conserved Ser/Thr protein kinase, operates in one of two protein complexes, mTOR complex 1 (mTORC1) and mTORC2 [17,18]. Of these, mTORC1 controls protein synthesis and may particularly enhance expression of cytoprotective proteins, thereby contributing to cell survival [19]. In contrast, AMPK is viewed as the master regulator of energy homeostasis, being activated by nutrient-poor conditions and increases in AMP associated with concomitant loss of ATP [16]. AMPK inhibits mTORC1 via inhibitory phosphorylation of an essential mTORC1 protein, Raptor, thus inhibiting protein synthesis. Redox stress inhibits protein synthesis in cardiomyocytes [20], but the mechanism is not fully understood.

Clearly, in cardiomyocytes (as in other cells), redox stress induced signalling has a range of effects on the cellular response and this could be due to a number of factors including effects of different forms of ROS [21] and/or localised ROS production to elicit redox signalling (e.g. from NADPH oxidases or mitochondria [[9], [10], [11]]). Here, we revisited the more fundamental hypothesis that the level of ROS stress per se is a key factor in the cellular response. We applied different concentrations of H_2_O_2_ (as the most physiologically relevant form of ROS) globally to cardiomyocytes, removing questions surrounding specific forms of stressors or subcellular localisation. We focussed on the AMPK-mTOR signalling axis and assessed the impact on expression of immediate early genes (IEGs) and protein synthesis. We detected increased mTOR signalling and induction of cytoprotective proteins such as p21^Cip1/WAF1^ with lower levels of oxidative stress, and inhibition of protein synthesis with higher concentrations associated with cell death. Thus, quite apart from considerations of ROS species and localisation, a “redox rheostat” effect operates in cells to elicit different responses according to the degree of stress.

## 2 Materials and methods

### 2.1 Cardiomyocyte isolation and culture conditions

Neonatal rat ventricular myocytes were dissociated from the ventricles of 2 to 4 day Sprague-Dawley rat hearts as described previously [22]. Cardiomyocytes were plated on Primaria culture dishes precoated with 1% (v/v) gelatin for 18 h in Dulbecco's modified Eagle's medium (DMEM)/M199 medium (4,1 ratio) containing 100 units/ml penicillin and streptomycin plus 15% (v/v) foetal calf serum (FCS) at 37 °C. Unless otherwise stated, cells were plated at a density of 4 × 10^6^ cells per 60 mm, 2 or 1.5 × 10^6^ (as indicated) cells per 35 mm dish, or 3 × 10^5^ cells/well in 24-well plates. Serum was withdrawn and cells incubated in maintenance medium (DMEM/M199; 24 h). Cells were exposed to H_2_O_2_ with or without 15 nM SU6656 (Calbiochem), 60 μM compound C (Calbiochem) or 1 μM KU63794 (Selleck Chemicals). These inhibitors were dissolved in DMSO and added directly to the medium (1/1000 dilution) before addition of H_2_O_2_.

### 2.2 Antisense oligodeoxynucleotide transfection

Serum-deprived cardiomyocytes in 35 mm Primaria dishes were exposed to phosphorothioate fluorescein-tagged antisense oligodeoxynucleotides (ODN, 200 nM; MWG, UK) corresponding to a sequence encompassing the initiation codon of p21^Cip1/WAF1^ (p21^Cip1/WAF1^AS, 5′_GACATCACCAGGATCGGACAT_3′) [23] or to 200 nM scrambled ODN (5′_TGGATCCGACATGTCAGA_3′) derived from the p21^Cip1/WAF1^ antisense (AS) sequence in serum-free DMEM containing 20 μg/ml lipofectin (Invitrogen). Cardiomyocytes were incubated for 24 h before addition of 0.2 mM H_2_O_2_ or 1 μM doxorubicin in DMSO.

### 2.3 Measurement of cardiomyocyte ATP concentrations

Cardiomyocytes in 24-well plates were incubated in 1 ml DMEM/M199 maintenance medium for 30 min before exposure to H_2_O_2_. Cells were scraped into 100 μl ice-cold 5% (v/v) perchloric acid and extracts centrifuged (10,000 ×*g*, 5 min, 4 °C). Supernatants were diluted 1/10 in KTME buffer [100 mM Tricine (pH 7.8), 10 mM MgCl_2_, 2 mM EDTA, 1 mM dithiothreitol] and duplicate samples (20 μl) were assayed. An ATP standard curve (linear over the range of ATP used) was constructed using ATP standards prepared in 5% (*v*/v) perchloric acid and diluted 1/10 in KTME buffer. Assays were initiated by addition of 100 μl KTME buffer containing 1% (v/v) firefly lantern extract (Sigma FLE-250, reconstituted at 10 mg/ml in PBS) and 75 μM luciferin, and luminescence measured using a TD-20/20 luminometer (Turner Designs) as light emitted in 10 s following a 3 s delay.

### 2.4 Measurement of protein synthesis

Cardiomyocytes (2 × 10^6^ cells per 35 mm dish) in maintenance medium were incubated with H_2_O_2_ (2 h) with addition of L-[2,3,4,5,6-^3^H]-phenylalanine ([^3^H]Phe, Perkin-Elmer) for the last 1 h of the incubation as published [24]. Cardiomyocytes were washed in ice-cold PBS (1 ml) and scraped into 0.2 mM NaOH (1 ml). A sample (15 μl) was taken to determine total protein using the Biorad Bradford method [25]. Bovine serum albumin (0.1 ml, 100 mg/ml) was added to the remaining samples as a carrier, proteins were precipitated with 10% (*w*/*v*) trichloroacetic acid (6 ml) and centrifuged. Precipitates were washed [10% (*w*/*v*) trichloroacetic acid (3 × 5 ml)], NaOH was added (10 μl, 10 M), and the pellets were dissolved in 1.8 ml Soluene (Perkin-Elmer, UK) before scintillation counting using Ultima Gold scintillation fluid (Perkin-Elmer, UK). Experiments were performed in duplicate, the mean values were taken and corrected according to total protein. The data are presented as means ± SEM of these values. IC_50_ values for each experiment were calculated using GraphPad Prism 7 software and the mean of these values was taken.

### 2.5 Immunoblotting

Cardiomyocyte cell lysates were prepared as described previously [22]. Briefly, cardiomyocytes were washed in ice-cold PBS and scraped into Buffer A [20 mM β-glycerophosphate pH 7.5, 20 mM NaF, 2 mM EDTA, 0.2 mM Na_3_VO_4_, 10 mM benzamidine, 5 mM dithiothreitol, 300 μM phenylmethylsulfonyl fluoride, 200 μM leupeptin, 2 μM microcystin LR, 10 μM trans-epoxy-succinyl-L-leucylamido-(4-guanidino)-butane] containing 1% (*v*/v) Triton X-100. Following centrifugation (10,000 ×*g*, 5 min, 4 °C), the supernatants were boiled with 0.3 vol sample buffer [10% (w/v) SDS, 13% (w/v) glycerol, 300 mM Tris-HCl pH 6.8, 130 mM dithiothreitol, 0.2% (w/v) bromophenol blue]. For isolation of the nuclear-enriched and cytosolic proteins, cells were processed as published [26]. Proteins were separated by SDS-polyacrylamide gel electrophoresis and transferred to nitrocellulose membranes. Proteins were detected with antibodies from Cell Signalling Technologies Inc. [phosphorylated AMPK(Thr^172^) (cat. no.: 2535), total AMPK (cat. no.: 2603), phosphorylated Raptor(Ser^792^) (cat. no.: 2083), total Raptor (cat. no.: 2280), phosphorylated mTOR(Ser^2448^) (cat. no.: 2971), phosphorylated mTOR(Ser^2481^) (cat. no.: 2974), total mTOR (cat. no.: 2983), phosphorylated PKB/Akt(Ser^473^) (cat. no.: 4060), phosphorylated PKB/Akt(Thr^308^) (cat. no.: 2965), total PKB/Akt (cat. no.: 4691), cleaved caspase 3 (cat. no.: 9664)] all used at 1/1000 dilution except for cleaved caspase 3 antibodies that were used at 1/500. Antibodies to p21^Cip1/WAF1^ were from Santa Cruz Biotechnology Inc. (cat. no.: sc-471; 1/1000 dilution) or Upstate Biotech (supplied by MERCK; cat. no. 05-345; 1/1000 dilution). Antibodies to sarcomeric α actin were from Sigma (cat. no.: A-2172; 1/1000 dilution). Proteins were detected by enhanced chemiluminescence using ECL Prime Western Blotting detection reagents with visualisation using an ImageQuant LAS4000 system (GE Healthcare). ImageQuant 7.0 software (GE Healthcare) was used for densitometry.

### 2.6 RNA isolation, microarray analysis, ratiometric PCR and qPCR

Total RNA was prepared using RNA Bee (AMS Biotechnology Ltd) according to the manufacturer's instructions. RNA was dissolved in nuclease-free water and purity was assessed from the A_260_/A_280_ with values of 1.8–2.1 (being considered acceptable). RNA concentrations were determined from the A_260_. For microarray analysis, total RNA (4 × 10^6^ cells per sample) and polysomal RNA (16 × 10^6^ cells per sample) were prepared from control cardiomyocytes or cardiomyocytes exposed to 0.2 mM H_2_O_2_ (1 h) as described in [24]. RNAs were prepared from 12 separate preparations of cardiomyocytes and individual RNA samples were generated by combining equal amounts of RNA from three separate myocyte preparations. Four sets of samples were hybridised to individual Affymetrix rat genome 230 2.0 microarrays. cRNA preparations and microarray hybridizations were performed as previously described [27]. Data (as CEL files) are available from ArrayExpress (accession number: E-MTAB-6758).

The CEL files were imported into GeneSpring GX13 for analysis using MAS 5.0 summarisation. Data were normalised to the gene median and a confidence filter applied (>50 raw value). Probesets were selected according to relative level of expression (>1.5-fold increase induced by H_2_O_2_ in total or polysomal RNA pools relative to controls) followed by statistical testing (one-way ANOVA with SNK post-test) using the Benjamini and Hochberg correction for multiple testing [false discovery rate (FDR) < 0.05]. To identify mRNAs with differential recruitment to polysomes, data for each RNA pool were normalised to the median of the control values. Probesets were selected with >1.5-fold change induced by H_2_O_2_ in polysomes relative to total RNA followed by statistical testing using a one-way ANOVA with SNK post-test and with Benjamini and Hochberg FDR < 0.05.

First strand cDNA synthesis with oligo-dT priming and ratiometric RT-PCR were performed as previously described [28] using primers for p21^Cip1/WAF1^ (F1, 5′_ACTTTGACTTCGCCACTGAG_3′; F2, 5′_CTGCTACAGTGCCCGAGTTA_3′; F3, 5′_TCGTGGTACGGATCAGTGAT_3′; R1, 5′_ACAGCAGAAGAAGGCGAGC_3′; R3, 5′_CAGAAGCGAGCTCTCGGTA_3′), or glyceraldehyde 3-phosphate dehydrogenase (GAPDH) (sense primer: 5′-ACCACAGTCCATGCCATCAC-3′; antisense primer: 5′-TCCACCACCCTGTTGCTGTA-3′; 418 bp product). Samples were heated (95 °C, 3 min) and subjected to 22 or 28 cycles of denaturation (95 °C, 50 s), annealing (61 °C, 50 s), and extension (72 °C, 50 s). Products were analyzed by ethidium bromide-agarose gel electrophoresis [2% (*w*/*v*) agarose gels] and the bands captured under UV illumination. Primer sets generated single products of the predicted sizes. Products were analyzed by scanning densitometry and normalised to GAPDH mRNA levels.

Quantitative PCR (qPCR) analysis was performed as previously described [27]. Total RNA was reverse transcribed to cDNA by using High Capacity cDNA Reverse Transcription Kits with random primers (Applied Biosystems) according to the manufacturer's instructions. qPCR was performed using an ABI Real-Time PCR 7500 system (Applied Biosystems). Optical 96-well reaction plates were used in parallel with the iTaq Universal SYBR Green Supermix (Bio-Rad Laboratories Inc.) according to the manufacturer's instructions. Primers were from Eurofins [Atf3 (NM_012912.1): sense primer: 5′-TCGCCATCCAGAACAAGCA-3′, antisense primer: 5′-GGGCCACCTCAGACTTGGT-3′, 108 bp product; Cdkn1a (NM_080782.3): sense primer: 5′- CGGGACCGGGACATCTC-3′, antisense primer: 5′- GGCACTTCAGGGCTTTCTCTT-3′, 106 bp product; Egr1 (NM_012551): sense primer: 5′- ACAACCCTACGAGCACCTG-3′, antisense primer: 5′- GGATAACTTGTCTCCACCAG-3′, 84 bp product; Gapdh (NM_017008): sense primer: 5′- GCTGGCATTGCTCTCAATGACA-3′, antisense primer: 5′- TCCACCACCCTGTTGCTGTA-3′, 83 bp product; *Hmox1* (NM_012580.2): sense primer: 5′- GACAGAGGAACACAAAGACCAGAGT-3′, antisense primer: 5′- GGTAGTATCTTGAACCAGGCTAGCA-3′, 82 bp product; Jun (NM_021835): sense primer: 5′- GATCATCCAGTCCAGCAATG-3′, antisense primer: 5′- TATTCTGGCTATGCAGTTCAG-3′, 140 bp product; *Mdm2* (NM_001108099.1): sense primer: 5′- TCCGACCACCGTGCTTCT-3′, antisense primer: 5′- TCGGTAGACACAGACATGTTGGTA-3′, 69 bp product; *Nfil3* (NM_053727): sense primer: 5′- TGGGTCACAGCCATCCGTT-3′, antisense primer: 5′- GCTTCAGCTTCTCGAATCCA -3′, 122 bp product; *Nqo1* (NM_017000.3): sense primer: 5′- GACATCACAGGGGAGCCG-3′, antisense primer: 5′- CTCAGGCGGCCTTCCTTATAC-3′, 83 bp product; *Rasd1* (XM_340809.4): sense primer: 5′- GCGGCGAAGTCTACCAGTTG-3′, antisense primer: 5′- AAAACGTCTCCTGTGAGGATAGAGA-3′, 94 bp product]. Relative quantification was obtained using a standard curve. Results were normalised to Gapdh mRNA levels, then to controls or zero time.

### 2.7 Statistics

Data are presented as mean ± SEM of the results from at least 4 independent myocyte preparations as detailed in the figure legends. Statistical analysis used one-way or two-way ANOVA with post-tests as indicated (GraphPad Prism 7 software). Results were considered statistically significant with *p* < .05.

## 3 Results

### 3.1 Redox stress reduces ATP concentrations and the rate of protein synthesis

**Fig. 1:**
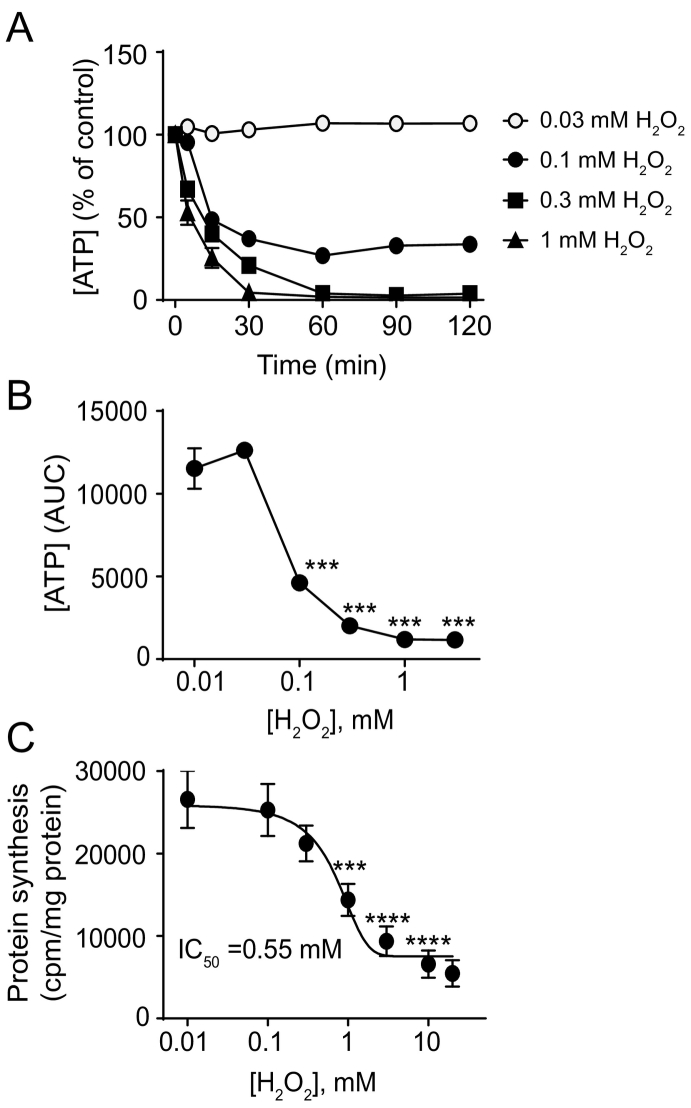
H_2_O_2_ reduces ATP levels and inhibits protein synthesis in cardiomyocytes. Cardiomyocytes were exposed to the concentrations of H_2_O_2_ shown for the times indicated. (A) Concentrations of ATP were measured using a luciferase assay. Results are % of control values and are means ± SEM (*n* = 4 independent myocyte preparations). (B) Area under curve (AUC) analysis of cardiomyocyte ATP levels from the data in (A). ***p < .001 relative to control (one-way ANOVA with Tukey post-test). (C) Protein synthesis was measured by incorporation of [^3^H]-Phe. Results are means ± SEM (*n* = 4 independent myocyte preparations). The IC_50_ was calculated using the 4-step parameter function in GraphPad Prism 7. ***p < .001, ****p < .0001 relative to 0.1 mM H_2_O_2_ (one-way ANOVA with Tukey post-test).

AMPK signalling, protein synthesis and apoptosis are intimately linked to intracellular ATP concentrations [16]. To explore this relationship in the context of redox stress induced signalling, we examined the effects of H_2_O_2_ (a physiologically-relevant form of ROS) on intracellular ATP concentrations in neonatal rat ventricular myocytes (Fig. 1A). These cells are cultured from 2 to 4-day old rats to ensure that the cells are post-mitotic. Though not completely representative of an adult cardiomyocyte, they are an appropriate model for assessment of general concepts of intracellular signalling in terminally-differentiated cardiomyocytes. At H_2_O_2_ concentrations ≥0.1 mM, ATP was rapidly lost (>50% reduction within 15 min). At ≥1 mM H_2_O_2_, intracellular ATP was essentially completely lost within 30 min, whereas at 0.1 mM H_2_O_2_, ATP concentrations were reduced by 60–70% at 30 min but thereafter there was no further decrease (Fig. 1A). Integration of the data in Fig. 1A showed that intracellular ATP concentrations were only significantly affected above 0.03–0.1 mM H_2_O_2_ with 50% of ATP being lost over 2 h with ~0.08 mM H_2_O_2_ (Fig. 1B). Protein synthesis requires ATP. Increasing concentrations of H_2_O_2_ decreased cardiomyocyte protein synthesis rates over 2 h with an IC_50_ of ~0.55 mM (Fig. 1C). This was despite the fact that about 90% of the intracellular ATP content was lost at this concentration of H_2_O_2_ (Fig. 1A). This suggests that the rate of global protein synthesis is relatively resistant to loss of ATP.

### 3.2 Concentration-dependent effects of H_2_O_2_ on phosphorylation and activation of AMPK, mTOR and PKB/Akt, and on phosphorylation of the mTORC1 component raptor

**Fig. 2:**
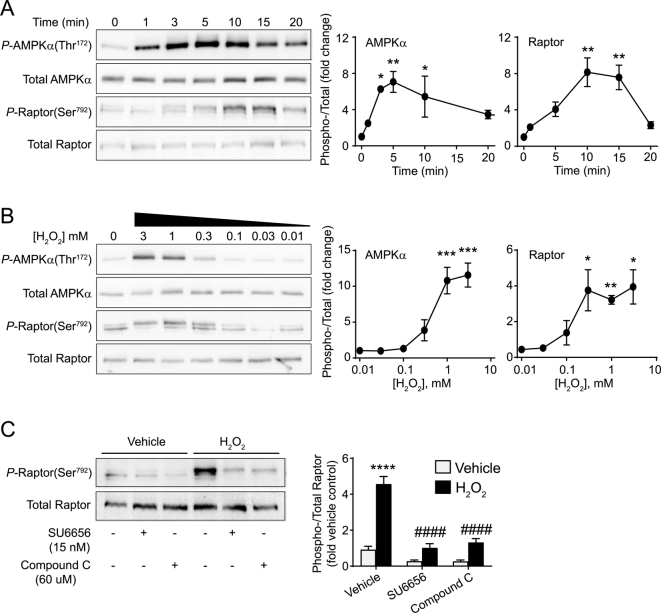
H_2_O_2_ activates AMPK and promotes AMPK-dependent phosphorylation of Raptor. Cardiomyocytes were exposed to 1 mM H_2_O_2_ for the times shown (A), to the concentrations of H_2_O_2_ indicated for 5 min (B), or were treated with SU6656 (15 nM) or compound C (60 μM) prior to addition of H_2_O_2_ (1 mM; 10 mins) (C). Samples were immunoblotted with antibodies to phosphorylated (*P*)-AMPKα(Thr^172^), total AMPKα, *P*-Raptor(Ser^792^) or total Raptor as indicated. Representative blots are shown in the left panels, with densitometric analysis and the ratio of Phospho−/Total proteins in the graphs on the right. (A) and (B), Results are means ± SEM (*n* = 4 independent myocyte preparations). *p < .05, **p < .01, ***p < .001 relative to unstimulated cells (one-way ANOVA with Dunnett's post-test). (C) Results are means ± SEM (*n* = 3 independent myocyte preparations). ****p < .0001 relative to unstimulated cells, #### *p* < .0001 relative to H_2_O_2_ alone (one-way ANOVA with Bonferroni post-test).

AMPK is a heterotrimer comprising a catalytic α subunit, a regulatory glycogen-binding β subunit, and a regulatory γ subunit which binds adenine nucleotides, and detects increases in AMP and, to a lesser extent, ADP [16]. It has been termed an ‘energy sensor’ or ‘intracellular fuel gauge’, becoming activated as ATP concentrations fall, and AMP (and ADP) concentrations rise. AMPK is activated by phosphorylation of AMPKα(Thr^172^), and decreases rates of anabolic processes (e.g. protein synthesis) whilst increasing rates of catabolic processes (e.g. lipolysis). Activation of AMPK by H_2_O_2_ in cardiomyocytes was assessed by immunoblotting of extracts for AMPKα(Thr^172^) phosphorylation. As in other cells [29], AMPKα(Thr^172^) was rapidly phosphorylated in response to 1 mM H_2_O_2_ (maximal within 3–5 min) (Fig. 2A) and the concentration-dependence was such that activation was detectable at 0.3 mM H_2_O_2_ and plateaued at 1 mM H_2_O_2_ (Fig. 2B).

mTORC1 regulates protein synthesis [17,18]. Raptor is a key regulatory subunit of mTORC1, and AMPK phosphorylates Raptor(Ser^792^), causing it to dissociate from mTORC1, thus inhibiting mTORC1 activity. Consistent with activation of AMPK, H_2_O_2_ increased phosphorylation of Raptor(Ser^792^) (Fig. 2A). This was delayed relative to AMPK activation, but the dependence on H_2_O_2_ concentration was similar (Fig. 2B). Compound C is an AMPK inhibitor, though it inhibits a number of other protein kinases [30]. SU6656 was originally identified as an inhibitor of the protein Tyr-kinase Src [31], but inhibits a number of other protein Ser-/Thr- kinases including AMPK [30]. Both compound C (60 μM) and SU6656 (15 nM) inhibited phosphorylation of Raptor(Ser^792^) induced in cardiomyocytes by H_2_O_2_ (1 mM, 10 min) (Fig. 2C). We conclude that H_2_O_2_ reduces cardiomyocyte ATP concentrations and this activates AMPK leading to phosphorylation of Raptor(Ser^792^). This is consistent with inhibition of protein synthesis (Fig. 1C).

### 3.3 H_2_O_2_ induces PKB/Akt and mTOR phosphorylation

**Fig. 3:**
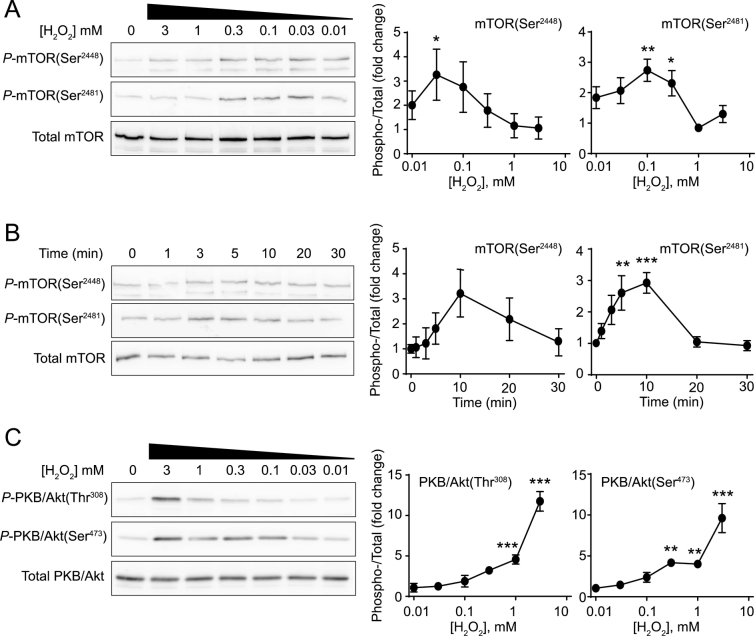
H_2_O_2_ (0.03–0.3 mM) activates mTOR but concentrations > 1 mM are required for activation of PKB/Akt. Cardiomyocytes were exposed to the concentrations of H_2_O_2_ indicated for 15 min (A) and (C), or to 0.2 mM H_2_O_2_ for the times shown (B). Samples were immunoblotted with antibodies to phosphorylated (*P*)-mTOR(Ser^2448^), *P*-mTOR(Ser^2481^), total mTOR, *P*-PKB/Akt(Ser^473^), *P*-PKB/Akt(Thr^308^) or total PKB/Akt as indicated. Representative blots are shown in the panels on the left, with densitometric analysis and the ratio of Phospho−/Total proteins in the graphs on the right. Results are means ± SEM [*n* = 4 (A) and (B) or *n* = 3 (C) independent myocyte preparations]. *p < .05, **p < .01 and ***p < .001 relative to unstimulated cells (one-way ANOVA with Dunnett's post-test).

mTOR, the protein kinase component of mTORC1, is itself phosphorylated at several sites including mTOR(Ser^2448^) and mTOR(Ser^2481^). mTOR(Ser^2448^) phosphorylation probably represents a downstream ‘feedback’ phosphorylation whereas mTOR(Ser^2481^) phosphorylation is an autophosphorylation that occurs when mTOR is activated [32,33]. In cardiomyocytes, phosphorylation of both sites was increased by low concentrations of H_2_O_2_ (0.03–0.2 mM), whereas higher concentrations (1–10 mM) reduced mTOR phosphorylation (Fig. 3A and B). Canonically, mTOR is activated by PKB/Akt [17,18]. PKB/Akt is phosphorylated on Thr^308^ in its catalytic domain and this is required for mTOR activation. It is also phosphorylated on Ser^473^ in its C-terminal hydrophobic domain with phosphorylation of both residues probably necessary for maximal activation [34]. Concentrations of H_2_O_2_ > 0.3 mM resulted in phosphorylation of both PKB/Akt(Thr^308^) and PKB/Akt(Ser^473^) (Fig. 3C). This should ultimately induce phosphorylation of mTOR(Ser^2448^) and mTOR(Ser^2481^), but there was no correlation with these phosphorylations (Fig. 3A and B). This implies that a supervening intervention, presumably by phosphorylation of AMPK and Raptor (Fig. 2A–C), interferes with PKB/Akt-mediated phosphorylation of mTOR(Ser^2448^) and mTOR(Ser^2481^).

### 3.4 Redox signalling vs. stress and expression of IEGs

**Fig. 4:**
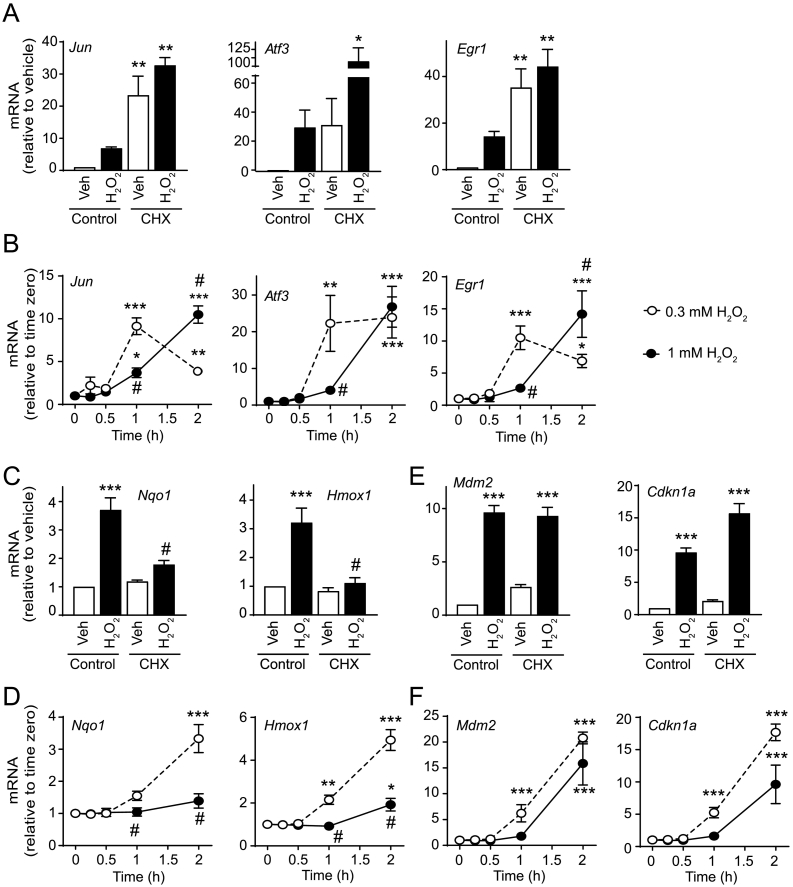
H_2_O_2_ upregulates immediate early genes, but not second-phase gene products. (A), (C) and (E) Cardiomyocytes were exposed to 0.2 mM H_2_O_2_ for 2 h in the absence or presence of 20 μM cycloheximide (CHX). (B), (D) and (F) Cardiomyocytes were exposed to 0.3 mM or 1 mM H_2_O_2_ for the times indicated. mRNA expression was measured by qPCR. Results are means ± SEM (*n* = 4–6 independent myocyte preparations). (A), (C) and (E) *p < .05, **p < .01 and ***p < .001 relative to unstimulated controls, # *p* < .05 relative to H_2_O_2_ alone (one-way ANOVA with Tukey post-test). (B), (D) and (F) *p < .05, **p < .01 and ***p < .001 relative to unstimulated controls, # *p* < .05 relative to 0.3 mM H_2_O_2_ (one-way ANOVA with Tukey post-test).

Expression of IEG mRNAs is driven by pre-existing transcription factors and de novo protein synthesis is not required [35]. This is characterised by an increase in mRNA expression in the presence of protein synthesis inhibitors such as cycloheximide. Indeed, because cycloheximide exerts an additional stress on the cell, IEG mRNAs are often superinduced. Many IEGs encode transcription factors which are required for expression of ‘later-phase’ genes and expression of these does require protein synthesis [36]. It may therefore be expected that high concentrations of H_2_O_2_ that inhibit protein synthesis do not affect IEG mRNAs but suppress expression of later-phase mRNAs. *Jun*, *Atf3* and *Egr1* are well-established IEGs. All were upregulated in cardiomyocytes exposed to 0.3 mM H_2_O_2_ (2 h) and this was enhanced by the protein synthesis inhibitor cycloheximide (Fig. 4A), confirming they are IEGs. As expected, these mRNAs were still upregulated by 1 mM H_2_O_2_, a concentration that substantially inhibits protein synthesis (Fig. 1C), although upregulation was delayed relative to 0.3 mM H_2_O_2_ (Fig. 4B). In contrast, upregulation of *Nqo1* and *Hmox1* by 0.3 mM H_2_O_2_ was inhibited by cycloximide (Fig. 4C), confirming they are later phase genes. These mRNAs were not significantly upregulated by 1 mM H_2_O_2_ (Fig. 4D). Our previous work identified *Mdm2* and *Cdkn1a* as genes which were significantly upregulated by low, sub-toxic concentrations of H_2_O_2_ (0.04 mM) [12] and we subsequently showed that *Mdm2* is cytoprotective under conditions of oxidative stress. [37]. Experiments with cycloheximide indicate that *Mdm2* and *Cdkn1a* were both upregulated as IEGs (Fig. 4E). Consistent with this, they were both upregulated by a high concentration of H_2_O_2_ (1 mM), albeit with some delay relative to 0.3 mM H_2_O_2_ (Fig. 4F) as was seen with *Jun*, *Atf3* and *Egr1* (Fig. 4B).

### 3.5 Polysomal profiling of mRNA transcripts

**Fig. 5:**
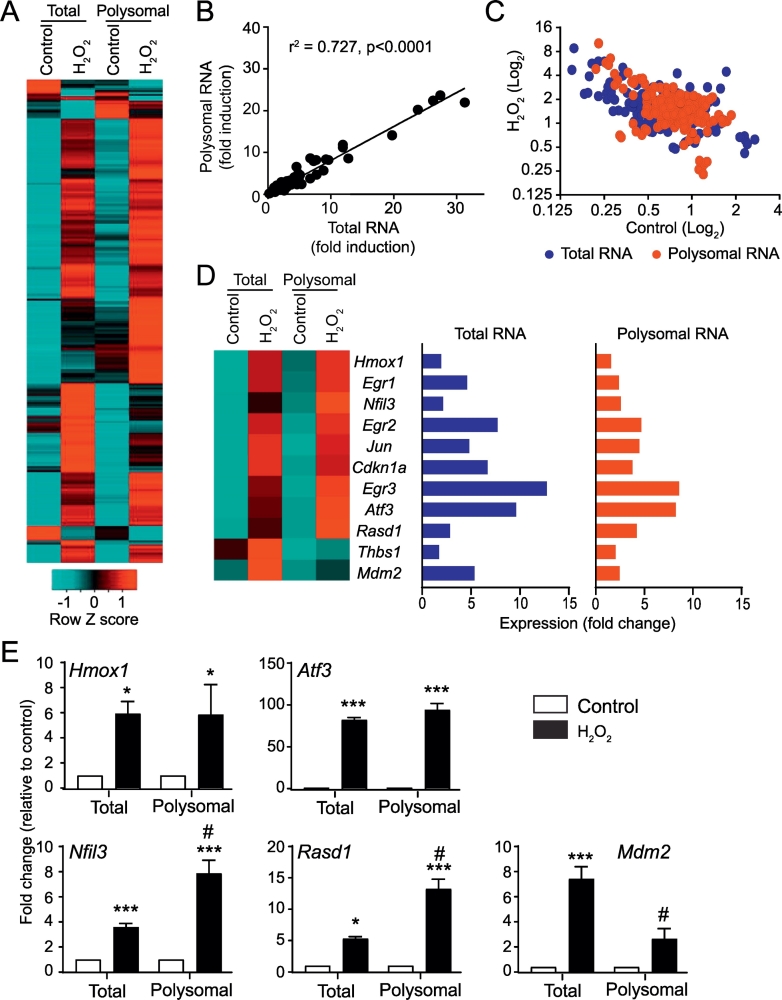
Recruitment of mRNAs induced by H_2_O_2_ to polysomes for translation. Cardiomyocytes were unstimulated (Control) or exposed to 0.2 mM H_2_O_2_ (1 h) and total or polysomal RNA prepared for microarray analysis [(A) - (D)] or qPCR (E). (A) Heatmap (Log_2_ scale) of all probesets with significantly decreased or increased expression (>1.5-Fold change; FDR < 0.05) in total or polysomal RNA fractions. Expression values were normalised to the gene median. (B) Relative fold change of upregulated mRNAs in polysomal vs total RNA pools. Linear regression analysis indicates that most mRNAs are regulated similarly in total and polysomal fractions and are translated efficiently. (C) Overlay plot of mRNAs induced by H_2_O_2_ in total and polysomal RNA pools to show differential changes in some genes. (D) Heatmap (Log_2_ scale) of specific genes relevant to this study is shown on the left, with fold change relative to controls on the right. (E) qPCR validation of microarray data showing similar changes in expression in total or polysomal RNA fractions for *Hmox1* and *Atf3*, enhanced recruitment of *Nfil3* and *Rasd1* to polysomes for translation and less efficient recruitment of *Mdm2* to polysomes. Results are means ± SEM (*n* = 4 independent myocyte preparations; these are different preparations from those used for microarray analysis). *p < .05, ***p < .001 relative to unstimulated controls, # *p* < .05 relative to fold-change in total RNA pool (one-way ANOVA with Tukey post-test).

Even though moderate concentrations of H_2_O_2_ (0.1–0.3 mM) did not suppress global protein synthesis to any significant extent (Fig. 1C), the substantial effect on ATP concentrations (Fig. 1A) could affect synthesis of specific proteins. We therefore examined recruitment of mRNAs to cardiomyocyte polysomes following exposure to 0.2 mM H_2_O_2_ (1 h) using Affymetrix microarrays for global transcriptomic profiling. At this early time, 26 probesets (18 established protein-coding mRNAs) were significantly downregulated with 238 probesets (162 established protein-coding mRNAs) significantly upregulated (>1.5-fold change, *p* < .05 FDR) in either the total or polysomal pool (Fig. 5A; Supplemental Spreadsheet 1). Of the upregulated mRNAs, 144 (e.g. *Atf3*, *Jun*, *Hmox1*) were not differentially regulated in the total pool relative to the polysomes (Supplemental Spreadsheet 1) with a strong positive correlation between the relative upregulation in each of the mRNA pools (Fig. 5B). However, the profiles were not entirely overlapping (Fig. 5C) and 12 mRNA species (*Anxa1, Egr1, Egr2, Egr3, Tp53inp1,Ereg, Hspa1, Tacr2, Mdm2, Txnrd1, Isg20,* and *Cdkn1a*) were upregulated to a significantly greater extent (>1.5-fold; FDR < 0.05) in the total pool relative to the polysomes suggesting that their polysomal recruitment was reduced. In contrast, there was preferential recruitment of four mRNA species (including *Rasd1*, *Clk1*, *Nfil1* and *Hist2h2aa3*) to the polysomes (Supplemental Spreadsheet 1). Others, such as *Thbs1* were differentially regulated, but the difference was either not statistically significant or below our 1.5-fold threshold. Examples of genes with differential expression are shown in Fig. 5D. The data for *Hmox1*, *Atf3*, *Nfil1*, *Rasd1* and *Mdm2* were validated by qPCR (Fig. 5E). Thus, even though ATP concentrations were substantially reduced (Fig. 1A), polysomal recruitment of ~90% of mRNA species was unaffected.

### 3.6 p21^Cip1/WAF1^ (the protein product of the Cdkn1a gene) induced by sub-toxic H_2_O_2_ levels is cytoprotective

**Fig. 6:**
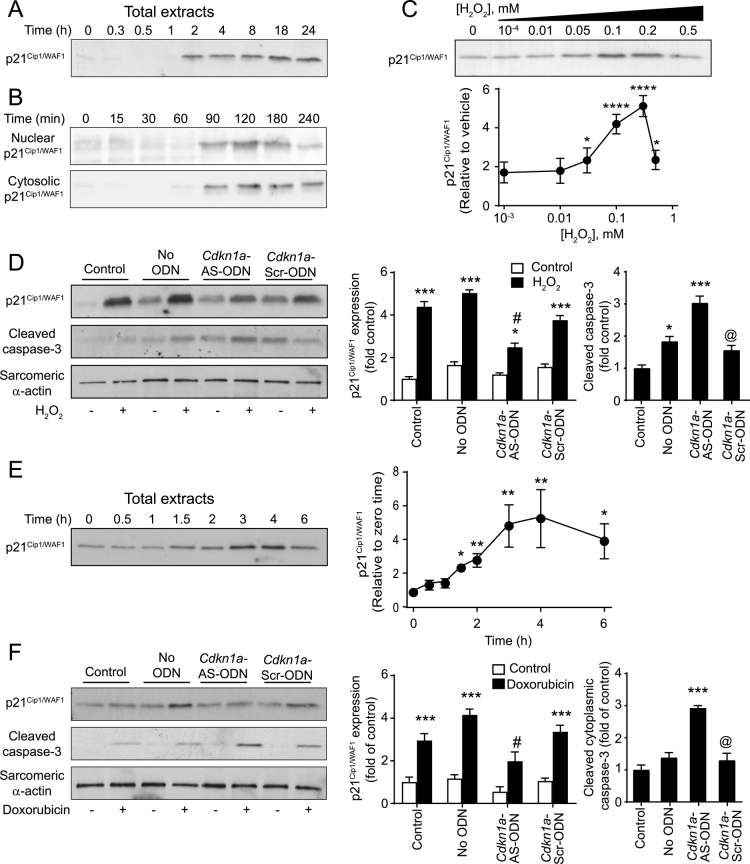
H_2_O_2_ or doxorubicin increase p21^Cip1/WAF1^ protein in cardiomyocytes to reduce apoptosis. (A) and (B) Cardiomyocytes were exposed to 0.2 mM H_2_O_2_ [(A) and (B)] or to 0.4 mM doxorubicin (E) for the times indicated and total extracts [(A) and (D)] or nuclear and cytosolic extracts (B) immunoblotted with antibodies to p21^Cip1/WAF1^. Representative immunoblots of at least 4 independent experiments are shown. Densitometric analysis is included to the right panels (A) and (D). Results are means ± SEM (*n* = 4 independent myocyte preparations). *p < .05, **p < .01 relative to unstimulated cells (one-way ANOVA with Dunnett's post-test). (C) Cardiomyocytes were exposed to the concentrations of H_2_O_2_ indicated for 120 min and immunoblotted with antibodies to to p21^Cip1/WAF1^. Densitometric analysis is included below the panel. Results are means ± SEM (*n* = 4 independent myocyte preparations). *p < .05, ****p < .0001 relative to unstimulated cells (one-way ANOVA with Dunnett's post-test). (D) and (F), Cardiomyocytes were exposed to 0.2 mM H_2_O_2_ (4 h) or 0.4 mM doxorubicin (4 h) alone (Control), following treatment with transfection reagent without oligodeoxynucleotides (no ODN) or following transfection with antisense ODNs (AS-ODNs) for *Cdkn1a* or scrambled ODNs (Scr-ODNs). Samples were immunoblotted with antibodies to p21^Cip1/WAF1^, cleaved caspase 3 or the loading control sarcomeric α-actin. Representative immunoblots are shown on the left, with densitometric analysis on the right. Data are means ± SEM (*n* = 6 independent myocyte preparations). *p < .05, ****p < .0001 relative to vehicle, #*p* < .01 relative to H_2_O_2_ treated cells with no ODNs, @ *p* < .05 relative to *Cdkn1a*-AS-ODN (two-way ANOVA with Tukey post-test).

p21^Cip1/WAF1^ was identified as a cyclin-dependent kinase inhibitor [38], but has other roles according its subcellular localisation [39,40]. Our previous microarray studies identified *Cdkn1a* as one of a small group of genes that was upregulated in cardiomyocytes by subtoxic concentrations of H_2_O_2_ [12], whilst here we have confirmed it is an IEG (Fig. 4E and F). However, the functional role of p21^Cip1/WAF1^ in cardiomyocytes is unknown. *Cdkn1a* mRNA was similarly upregulated in total and polysomal RNA pools (Fig. 5D), indicating that it is likely to be translated. Consistent with this, p21^Cip1/WAF1^ protein was rapidly increased in cardiomyocytes exposed to 0.2 mM H_2_O_2_ with maximal expression from ~2 h that was sustained up to at least 24 h (Fig. 6A). p21^Cip1/WAF1^ protein was detected in both nuclear and cytosolic extracts from ~90 min (Fig. 6B). Interestingly, the concentration-dependency of the response was bell-shaped and p21^CIP1/WAF1^ was not induced by 0.5 mM H_2_O_2_ at 2 h (Fig. 6C). This presumably reflects the reduced rate of protein synthesis (Fig. 1C).

To determine if p21^Cip1/WAF1^ is cytoprotective in cardiomyocytes, cardiomyocytes were transfected with *Cdkn1a* antisense oligonucleotides (*Cdkn1a*-AS-ODN) to suppress p21^Cip1/WAF1^ protein expression or a scrambled version of the *Cdkn1a*-AS-ODN (*Cdkn1a*-Scr-ODNs). As expected, *Cdkn1a* AS-ODN accumulated in the nucleus (not shown). H_2_O_2_ (0.2 mM, 4 h) increased expression of p21^Cip1/WAF1^ protein in transfected cells (Fig. 6C). This was significantly inhibited by *Cdkn1a-*AS-ODN (52 ± 4.6% relative to H_2_O_2_ alone, *p* < .01) but not by *Cdkn1a*-Scr-ODN. We demonstrated that 0.2 mM H_2_O_2_ induces apoptosis in neonatal rat cardiomyocytes as assessed by loss of mitochondrial membrane potential, TUNEL analysis and immunoblotting for cleaved caspase 3 [37], with detection of cleaved caspase 3 being probably the most reliable marker of cell death for quantificaiton purposes [44]. The reduction in p21^Cip1/WAF1^ protein by C*dkn1a-*AS-ODN in the context of H_2_O_2_ stimulation enhanced the amount of cleaved caspase 3, indicative of increased apoptosis (Fig. 6C). The anthracyclin doxorubicin is an anti-cancer agent that is cardiotoxic and increases ROS generation and promotes cardiac oxidative stress [41]. Doxorubicin increased expression of p21^Cip1/WAF1^ protein over 3–4 h (Fig. 6D). As with H_2_O_2_, this was significantly inhibited by *Cdkn1a-*AS-ODN but not by *Cdkn1a*-Scr-ODN, and decreased expression of p21^Cip1/WAF1^ protein was associated with enhanced appearance of cleaved caspase 3 and, thus, apoptosis (Fig. 6E). Hence, p21^Cip1/WAF1^ is cytoprotective in the cardiomyocyte response to redox/anthracycline stress.

### 3.7 mTOR regulates expression of p21^Cip1/WAF1^

**Fig. 7:**
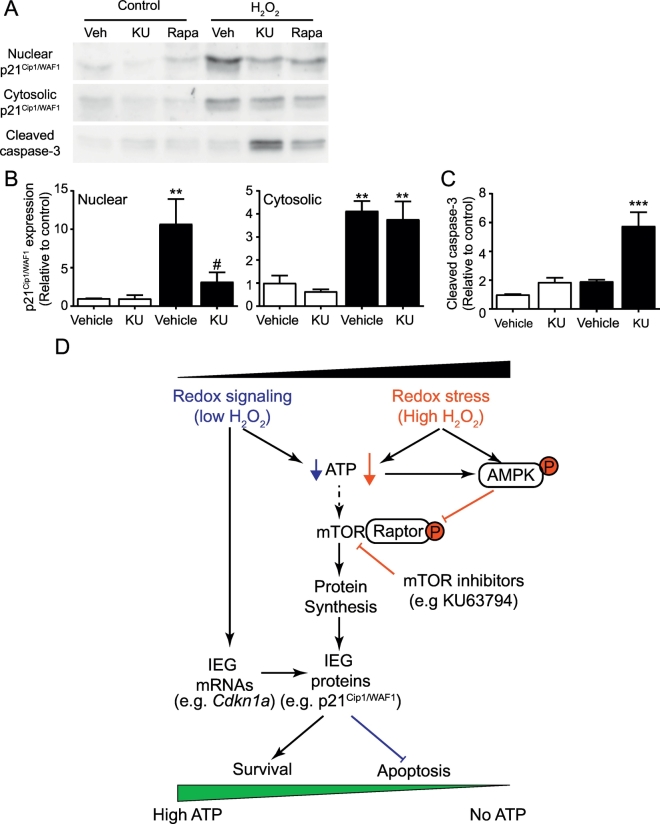
Inhibition of mTOR reduces the increase in expression of nuclear p21^Cip1/WAF1^ in cardiomyocytes induced by H_2_O_2_ and promotes apoptosis. (A) Cardiomyocytes were unstimulated (Control) or exposed to 0.2 mM H_2_O_2_ (2 h) in the presence of 1 μM KU63794, 10 μM Rapamycin or vehicle only. (B and C) Nuclear or cytosolic samples were immunoblotted with antibodies to p21^Cip1/WAF1^, [(A) and (B)] or cleaved caspase 3 [(A) and (C)]. Data are means ± SEM (*n* = 3 independent myocyte preparations); *p < .05, **p < .01, ***p < .001 relative to vehicle control; #*p* < .05 relative to H_2_O_2_ (one-way ANOVA with Tukey post-test). (D) Schematic for regulation of signalling and gene expression under conditions of redox stress vs signalling. In conditions of low H_2_O_2_, immediate early genes (IEGs) are upregulated and mTOR is activated to promote protein synthesis, leading to production of antioxidant enzymes and cytoprotective proteins such as p21^Cip1/WAF1^ to alleviate the stress of increased oxidative burden. Under conditions of high H_2_O_2_, ATP levels fall dramatically causing activation of AMPK with phosphorylation of Raptor, inhibition of mTORC1 and decreased protein synthesis. Cells are unable to synthesise cytoprotective proteins such as p21^Cip1/WAF1^ and undergo apoptosis.

mTOR is phosphorylated in cardiomyocytes exposed to moderate levels of H_2_O_2_ (0.1–0.3 mM) (Fig. 3A and B), indicative of its activation. The *pan*-mTOR inhibitor KU63794 [42] and mTORC1 inhibitor rapamycin almost completely prevented p21^Cip1/WAF1^ expression in the nuclear fraction (Fig. 7A) but had no effect on its expression in the cytoplasmic fraction (Fig. 7B). This was associated with increased caspase-3 cleavage (5.76 ± 0.9 fold) (Fig. 7C). Thus, mTOR is required for cytoprotection in the context of low levels of redox stress, in part by increasing expression of cytoprotective proteins such as p21^Cip1/WAF1^.

## 4 Discussion

Redox levels exert pleiotropic effects on cellular responses, ranging from promotion of cell survival through to regulation of cell death. Perhaps unsurprisingly, therefore, redox stress underlies a plethora of disease states including heart failure. Understanding how ROS signalling can elicit such a panoply of responses is of crucial importance for the development of novel therapies for these diseases. Various hypotheses have been developed to explain the variation of responses, including effects of different forms of ROS [21] and localised ROS production for redox signalling (e.g. from NADPH oxidases or mitochondria [[9], [10], [11]]). Whilst these factors clearly play a role, we assessed a more fundamental hypothesis that the level of ROS per se is a key factor in the cellular response, focusing on the AMPK-mTOR signalling axis, regulation of protein synthesis and cell death. We demonstrated the presence of a “redox rheostat” in which lower levels of ROS activate mTOR with production of cytoprotective proteins such as p21^Cip1/WAF1^, whilst higher levels cause a reduction in ATP with activation of AMPK, inhibition of protein synthesis and cell death (Fig. 7D). We propose that a sliding scale operates between these extremes, with variation in production of antioxidants and cytoprotective proteins balanced against ATP levels. This accounts for whether cells survive or grow and, if the balance is in favour of cell death, the mode of cell death in which highly regulated apoptosis will proceed if ATP levels suffice, but necrotic cell death occurs if there is catastrophic loss of ATP [43].

The relationship between intracellular ATP concentrations, and AMPK-mTOR signalling in relation to protein synthesis is clearly complex. Whilst it may seem obvious that declining ATP concentrations should be associated with inhibition of protein synthesis, the concentration-dependencies did not overlap fully, with substantial loss in global ATP at concentrations that did not appear to impinge on global protein synthesis (Fig. 1). Instead, significant inhibition of protein synthesis occurred at concentrations ≥1 mM H_2_O_2_, that are highly toxic to cardiomyocytes [44], and we suggest this represents complete shutdown of cellular processes whilst striving for survival. The mechanism most likely involves AMPK phosphorylation of Raptor(Ser^792^), a mechanism shown in other systems to inhibit global protein synthesis via inhibition of mTORC1 [[15], [16], [17], [18]], since the concentrations of H_2_O_2_ required to promote phosphorylation of AMPKα(Thr^172^) and Raptor(Ser^792^) (Fig. 2B) were those that caused inhibition of protein synthesis (Fig. 1C). Classically, mTOR (and therefore mTORC1) is activated by PKB/Akt [17,18], and we have shown previously that H_2_O_2_ activates PKB/Akt, increasing phosphorylation of both Thr^308^ and Ser^473^ [20]. Here, as before [20], PKB/Akt was only significantly phosphorylated on both residues with >1 mM H_2_O_2_ (Fig. 3C), concentrations that appear incompatible with protein synthesis (Fig. 1C). Our previous work identified an over-riding phosphatase activity causing dephosphorylation of 4E-BP1 to reduce availability of eIF4E for initiation of translation [20]. Here, the data suggest there is an additional mechanism with inhibition of mTOR via AMPK-dependent phosphorylation of Raptor(Ser^792^) (Fig. 2).

Whilst high concentrations of H_2_O_2_ inhibited mTORC1 and global protein synthesis, our data indicated that lower concentrations were associated with increased phosphorylation of mTOR(Ser^2448^) and mTOR(Ser^2481^) (Fig. 3A). The mechanism is unclear since we did not detect significant increases in PKB/Akt phosphorylation at these concentrations (Fig. 3C). This was not associated with a significant increase in global protein synthesis (Fig. 1C), but lower concentrations were associated with differential recruitment of some mRNAs to the polysomes of cardiomyocytes with enhanced recruitment (and therefore increased efficiency of translation) of some (e.g. *Nfil3*, *Rasd1*) and reduced efficiency of others (e.g. *Mdm2*) (Fig. 5). Indeed, these data are consistent with studies in brain in which focal ischaemia (associated with increased oxidative stress) results in selective recruitment of transcripts to the polysomes for translation [45]. As in the brain, and in contrast to insulin treatment of cardiomyocytes [24], we did not detect selective recruitment of transcripts with 5′ terminal oligopyrimidine tracts (TOPs) that are classically recruited following activation of p70 ribosomal S6 kinase via mTORC1.

The AMPK-mTOR axis is intimately linked to regulation of protein synthesis. However, the rate of global protein synthesis has a significant impact on expression of certain transcripts, namely those which require de novo synthesis of the transcription factors that regulate their expression. The concept of IEGs derived from early work on viral gene expression, but was extended to mammalian genes in which IEGs were defined as those for which transcription requires only pre-existing transcription factors [35]. This means that protein synthesis is not required, and inhibiting protein synthesis with, for example, cycloheximide, or H_2_O_2_ did not abolish upregulation of classic IEG mRNAs including *Jun, Atf3* and *Egr1* (Fig. 4A and B), although the rate of increase was delayed with 1 mM H_2_O_2_, possibly a reflection of the low energy status of the cells. These and many other IEGs encode transcription factors required for expression of later phase non-IEGs including *Hmox1*, and *Nqo1*, so if protein synthesis is inhibited and IEG mRNAs are not translated, these later phase genes are not upregulated even at the mRNA level. This was clearly apparent and upregulation of *Hmox1* and *Nqo1* mRNAs was abolished by cycloheximide or 1 mM H_2_O_2_ (Fig. 4C and D). These mRNAs along with other non-IEGs (e.g. *Gclc*, *Txnrd1*, *Srxn1*) form part of the neutralising response to an oxidant load, so inhibition of expression of these genes is likely to compromise cellular defences further, exacerbating the effects of high levels of redox stress and hasten progression to cell death.

This study, together with our previous microarray studies [12], identified a number of genes that are classically regulated by p53 including *Mdm2* and *Cdkn1a* (encoding p21^Cip1/WAF1^ protein). We have already reported that upregulation of *Mdm2* mRNA by H_2_O_2_ did not appear to require de novo synthesis of p53, with it being regulated by AP-1 transcription factors [37]. Here, we show that both *Mdm2* and *Cdkn1a* are upregulated by moderate concentrations of H_2_O_2_ (0.2 mM) in cardiomyocytes as IEGs (Fig. 4E and F). p21^Cip1/WAF1^ protein expression induced by 0.2 mM H_2_O_2_ was sustained over 2–24 h (Fig. 6A and B) and, as with *Mdm2* [37], preventing protein expression with antisense ODNs enhanced the rate of apoptosis shown by caspase-3 cleavage [37,44] (Fig. 6C), indicating that it is also a cytoprotective protein. This is consistent with a recent report in which p21^Cip1WAF1^ was shown to protect H9c2 cells (a cardiomyocyte-like cell line) in a simulated ischaemia-reperfusion injury model [46]. A role for p21^Cip1WAF1^ in cardiomyocyte cytoprotection is also consistent with reports in other cells in which the protective effect is attributed to accumulation in the cytoplasm rather than the nucleus [47]. In lung adenocarcinoma cells [48,49], cytosolic p21^Cip1WAFf1^ suppresses mitochondrial cell death pathways, including caspase-3 activation by preserving anti-apoptotic Bcl-2 family members. Based on our findings and previous work, we rationalise that the role for cytosolic p21^Cip1/WAF1^ in myocytes is to inhibit mitochondrial death pathways given that oxidative stress negatively alters Bcl-2 signalling [44], leading to cardiac myocyte apoptosis. However, p21^Cip1/WAF1^ was detected in both myocyte compartments (Fig. 6B), suggesting that it has an additional role in the nucleus even in terminally-differentiated post-mitotic cells. Interestingly, inhibition of mTOR with KU63794 or rapamycin partially inhibited the increase in p21^Cip1/WAF1^ protein induced by 0.2 mM H_2_O_2_, but inhibition was preferentially targeted to the nuclear compartment, with relative preservation of the protein in the cytoplasm (Fig. 7A and B). Nevertheless, both KU63794 and rapamycin enhanced the rate of cardiomyocyte apoptosis induced by 0.2 mM H_2_O_2_.

Our data here indicate that p21^CIP1/WAF1^ is protective in cardiomyocytes exposed to 0.2 mM H_2_O_2_, and previously we have shown that *Mdm2* also contributes to cytoprotection [37]. Nevertheless, cardiomyocytes still undergo apoptosis under these conditions as we have shown previously [44], indicating that the presence of these proteins is not sufficient for complete protection and raising the question of the importance of their upregulation. One consideration is that some degree of cytoprotection may be required to ensure that cell death proceeds via regulated programmed cell death and loss of protective elements such as expression of p21^CIP1/WAF1^ and *Mdm2* may increase the probability of non-regulated cell death (i.e. necrosis with associated inflammation). Another consideration is that we all study an entire population of cells and, for this study, each sample was prepared from 4 million cardiomyocytes. There is undoubtedly a spectrum of response and we measure the average. We simply do not know (and do not have the ability to assess) if these proteins are expressed only in a subpopulation of surviving cells. At higher concentrations of H_2_O_2_, protein synthesis is suppressed and, even though mRNAs increase, protein expression is compromised. Consistent with this, the concentration-dependency for expression of p21^CIP1/WAF1^ protein is bell-shaped with little induction of protein at 0.5 mM at 2 h (Fig. 6C). It remains to be established whether forced expression of proteins such as p21^CIP1/WAF1^ and *Mdm2* would confer cytoprotection under conditions of high level ROS, when cellular metabolism is compromised.

In summary, we present data to support a novel concept of the cardiomyocyte “redox rheostat” in which different degrees of ROS stress influence cell energetics and intracellular signalling pathways to regulate mRNA and protein expression. This sliding scale of responses determines cell fate, modulating cell survival vs cell death.

## Appendix A Supplementary data

Supplemental Spreadsheet 1: total vs. polynomial transcriptomic profiling. Image 1
